# A Resting State Functional Magnetic Resonance Imaging Study in Migraine Without Aura in Middle and High Altitude Areas

**DOI:** 10.1002/brb3.70975

**Published:** 2025-11-05

**Authors:** Jiahui Yao, Guangyi Yang, Guocai Yang, Yonghong Qi, Jinhao Liu, Yuhui Xiong, Yi Zhu, Xiaoli Yang, Qingxin Zhang

**Affiliations:** ^1^ Qinghai University Medical College Qinghai University Xining China; ^2^ Department of Magnetic Resonance Qinghai Provincial People's Hospital Xining China; ^3^ GE HealthCare MR Research Beijing China; ^4^ Philips Healthcare Xining China; ^5^ Department of Neurology Qinghai Provincial People's Hospital Xining China

**Keywords:** amplitude of low‐frequency fluctuations, functional magnetic resonance imaging, mid‐to‐high altitude, migraine without aura, regional homogeneity

## Abstract

**Objectives:**

This study used resting state functional magnetic resonance imaging (rs‐fMRI) technology to explore the characteristics of brain functional activity in migraine patients without aura (MwoA) in middle and high altitude areas during interictal periods through two analysis methods, the regional homogeneity (ReHo) and amplitude of low‐frequency fluctuation (ALFF).

**Methods:**

This study was a prospective research that included 41 patients with MwoA in the interictal phase, who visited the Department of Neurology at Qinghai Provincial People's Hospital between January 2023 and January 2024. 39 healthy controls (HCs) matched for age and sex were also recruited.

**Results:**

Compared with HCs group, the ALFF values of right superior temporal gyrus and the right hippocampus in MwoA group at mid‐to‐high altitude were decreased (voxel level *p* < 0.001, cluster level *p* < 0.05, Gaussian random field, GRF corrected). The ReHo values of bilateral rectus gyrus and left cerebellum in MwoA group at mid‐to‐high altitude were significantly increased, while the ReHo values of left cingulate gyrus, bilateral precuneus and bilateral supplementary motor area were significantly decreased (voxel level *p* < 0.001, cluster level *p* < 0.05, GRF corrected). The correlation analysis showed that the duration of disease in MwoA group was negatively correlated with the *z*‐ALFF value of the right hippocampus(*r* = −0.56, *p* = 0.004, Bonferroni correction). The HIT‐6 score was negatively correlated with the *z*‐ALFF value of the right superior temporal gyrus (*r* = −0.48, *p* = 0.001, Bonferroni correction). The SDS score was negatively correlated with the bilateral precuneus *z*‐ReHo values (*r* = −0.42, *p* = 0.03, L; *r* = −0.46, *p* = 0.01, R, Bonferroni correction).

**Conclusion:**

Several brain regions in MwoA patients from mid‐to‐high altitude areas exhibit abnormal spontaneous neural activity through ALFF and ReHo assessments. These brain regions are closely associated with pain processing, cognitive functions, motor control, attention, and emotional regulation. The functional abnormalities in these regions may be relevant to the pathophysiology of MwoA.

## Graphical abstract

A Resting‐state fMRI reveals altered functional connectivity within pain‐processing and default mode networks in migraine without aura patients residing at middle‐to‐high altitudes, which correlates with headache severity.

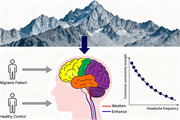

## 1 Introduction

Migraine is a prevalent neurological disorder characterized by moderate to severe pulsating headaches, often accompanied by sleep disturbances, nausea, vomiting, and increased sensitivity to light, sound, and other sensory stimuli, severely impacting patients’ quality of life (Puledda et al. 2024; Schwedt et al. 2015). In recent years, the global prevalence of migraine has increased significantly, affecting approximately 12% of the population worldwide (Vetvik and MacGregor 2017; Safiri et al. 2022).

Migraine without aura (MwoA) is the most common type of migraine, accounting for 70%–80% of all migraine cases. Despite advances in treatment, the management of this condition remains challenging, significantly affecting patients’ quality of life. Currently, there are inadequacies in the diagnosis of MwoA. For example, the American Migraine Prevalence and Prevention Study indicates that only 48% of respondents are satisfied with their diagnosis (Olesen 2024). In addition, MwoA is often accompanied by comorbidities such as stress and sleep disorders, further burdening patients (Scher et al. 2005).

Previous studies have shown that genetic factors and gender play important roles in the pathogenesis of migraine. It is estimated that the genetic probability of developing migraine is as high as 65%, with a significant familial aggregation observed (Pozo‐Rosich et al. 2025; Mulder et al. 2003; Villar‐Martinez and Goadsby 2024).

Recently, research has revealed that altitude may also influence the incidence and severity of migraine. The environmental conditions in medium to high‐altitude regions differ significantly from those at low altitudes. Key characteristics of these regions include hypoxia, dramatic temperature fluctuations, increased ultraviolet radiation, decreased humidity, and reduced atmospheric pressure. These environmental stressors pose significant physiological challenges to both temporary residents and local inhabitants. Several studies suggest that high‐altitude environments may impact the occurrence and development of migraines through multiple mechanisms. First, hypoxia can cause cerebral vasoconstriction, leading to reduced blood flow and oxygen supply, thereby triggering headaches. Second, pressure changes in high‐altitude regions may affect the dynamic balance of cerebrospinal fluid pressure, exacerbating headache symptoms. In addition, enhanced ultraviolet radiation and dramatic temperature changes are also considered important migraine triggers.

Several studies supported the association between high‐altitude environments and increased migraine prevalence. For instance, individuals living or traveling in high‐altitude areas have a significantly higher incidence of migraines compared to those in low‐altitude regions. Furthermore, people residing in high‐altitude regions often report more severe and longer‐lasting migraine symptoms (Linde et al. 2017; Ma et al. 2024; Serrano‐Duenas 2007; Arregui et al. 1991). This suggests that high‐altitude environments not only increase the risk of developing migraines but also exacerbate the severity of symptoms. In China, there are many high‐altitude regions with average elevations ranging from 3000 to 5000 m (Barry and Pollard 2003). However, there was still a significant gap in research and reports on MwoA patients living in these medium to high‐altitude areas.

Resting state functional magnetic resonance imaging (rs‐fMRI) provides a powerful tool for understanding brain function changes in MwoA patients in high‐altitude regions. As a non‐invasive technique, fMRI relies on low‐frequency fluctuations in blood oxygen level‐dependent (BOLD) signals to reveal brain activity patterns in a resting state. To date, numerous rs‐fMRI studies have uncovered brain function changes in migraine patients during rest, offering new insights into the pathogenesis of migraines (Pozo‐Rosich et al. 2025; Ma et al. 2024).

Therefore, this study aimed to use rs‐fMRI to preliminarily investigate the characteristic of brain functional activity in MwoA patients from mid to high altitude regions, providing imaging evidence for studying MwoA in these areas. This would help to deepen the understanding of the impact of high‐altitude environments on migraine patients and provide a scientific basis for developing more effective diagnostic and therapeutic strategies.

## 2 Materials and Methods

### 2.1 Study Subjects and Criteria

This study was a prospective research project that included 41 patients with MwoA in the interictal phase, all participants signed informed consent forms and voluntarily participated in the study. The research protocol was reviewed and approved by the Ethics Committee of our hospital (Approval No.2033‐077).

The inclusion criteria for the MwoA group were as follows: diagnosis confirmed by two senior neurologists based on the 2022 Chinese guidelines for migraine management and the 2018 Third Edition of the International Classification of Headache Disorders (ICHD‐3) criteria for MwoA; at least five headache attacks fulfilling criteria B–D, with attacks lasting 4–72 h (untreated or unsuccessfully treated), at least two of the following four characteristics: unilateral location, pulsating quality, moderate or severe pain intensity, aggravation by or causing avoidance of routine physical activity (e.g., walking or climbing stairs); and at least one of the following: nausea and/or vomiting, photophobia, and phonophobia. In addition, the altitude of the residences of all patients with migraines was within the range of 2200–4500 m.

Exclusion criteria included poor quality of MRI images, claustrophobia, or other MRI contraindications, significant head lesions detected on MRI, no use of migraine medication within 7 days before the MRI, other primary or secondary headaches such as tension‐type headache and cluster headache, and pregnancy or menstruation in women.

The inclusion criteria for the HCs group were: long‐term residents living at altitudes between 22004500 m, no significant head lesions based on medical records and clinical assessment, no history of relevant physical diseases, no family history of headaches or headache attacks within the past year, ability to understand and complete relevant scales, right‐handed, signed informed consent, voluntary participation in the study, and aged between 20 and 50 years. The exclusion criteria included poor quality of MRI images, claustrophobia, or other MRI contraindications, and pregnancy or menstruation in women.

### 2.2 Data Collection and Processing

#### 2.2.1 Clinical Data and Scale Collection

In this study, both MwoA group and HCs group completed the Self‐Rating Depression Scale (SDS), the Pittsburgh Sleep Quality Index (PSQI), and the Self‐Rating Anxiety Scale (SAS). In addition, the MwoA group completed the Migraine Disability Assessment Questionnaire (MIDAS) and the Headache Impact Test‐6 (HIT‐6). Assessments were conducted by two deputy chief physicians with appropriate clinical training and experience.

#### 2.2.2 Imaging Data Collection

All MRI scans were performed using a GE SIGNA Architect MRI 3.0T with a 64‐channel head coil. High‐resolution structural images were acquired using a 3D‐T1 Sag MP‐RAGE sequence with the following parameters: 150 slices, 1 mm slice thickness, 8° flip angle, repetition time (TR) of 2546 ms, echo time (TE) of 3.08 ms, and voxel size of 1 mm × 1mm × 1 mm, taking 5 min and 48 s. Resting‐state functional images were obtained using an echo‐planar imaging technique with the following settings: 7000 images, 35 slices, 3 mm slice thickness, no inter‐slice gap, 90° flip angle, 72 × 72 matrix, TR of 2000 ms, TE of 30 ms, over a duration of 6 min and 40 s.

### 2.3 Data Processing

#### 2.3.1 Data Preprocessing

Using the DPABI (Data Processing and Analysis for Brain Imaging) software package on the Matlab2017a platform, MRI data underwent preprocessing steps: (1) conversion of all DICOM images to NIFTI format; (2) removal of the initial 10 time points: to reduce effects of scanner instability; (3) slice timing; (4) head motion correction: exclude patients whose head motion exceeds 3 mm in any direction; (5) spatial normalization: the functional images of all the subjects were uniformly aligned into the MNI space using the diffeomorphic anatomical registration through exponentiated lie algebra (DARTEL) method; (6) linear detrending and covariate regression analysis.

#### 2.3.2 ALFF Analysis

Amplitude of low‐frequency fluctuation (ALFF) analysis was performed using DPABI. After preprocessing, data were band‐pass filtered between 0.01 and 0.08 Hz to exclude high‐frequency noise from the MRI and physiological noises such as heartbeat and respiration. Fast Fourier transform (FFT) was applied to compute the power spectral density, extracting the mean amplitude within the low‐frequency range as the ALFF value. ALFF maps were then smoothed using a 6 mm × 6 mm × 6 mm Gaussian kernel to enhance the signal‐to‐noise ratio. Finally, the obtained ReHo values were transformed into *z*‐score for subsequent statistical analysis.

#### 2.3.3 ReHo Analysis

Regional Homogeneity (ReHo) analysis was also conducted using DPARSF. The Kendall's coefficient of concordance was used to measure the synchrony of time series between each voxel and its 26 neighbors, reflecting the functional connectivity strength within brain regions. ReHo maps were smoothed with a 6 mm full‐width at half‐maximum (FWHM) Gaussian kernel to reduce spatial noise. Finally, the obtained ReHo values were transformed into *z*‐score for subsequent statistical analysis.

### 2.4 Correlation Analysis

This study extracted the numerical values of the brain regions with differences in ALFF and ReHo in patients with migraine, and conducted correlation analyses between these values and the clinical characteristics of the migraine patients. The correlation analysis needed to be corrected by Bonferroni. If *p* value was less than 0.05, it was considered that the correlation was statistically significant.

### 2.5 Statistical Analysis

Statistical analyses were carried out using SPSS 27.0, where normally distributed variables (e.g., SDS, PSQI, SAS, HIT‐6 scores, disease duration, and age) were described using mean ± standard deviation and analyzed using independent samples *t*‐tests. Skewed data (e.g., educational years and MIDAS scores) were described using medians and quartiles, with rank‐sum tests for educational years and Chi‐square tests for gender. Significance was set at *p* < 0.05. For functional imaging data, ReHo, and ALFF values calculated by DPABI were statistically analyzed considering covariates such as sex, age, education, and head motion parameters (Mean Framewise Displacement), using independent *t*‐tests with multiple comparison corrections (voxel level *p* < 0.001, cluster level *p* < 0.05, two tailed) based on Gaussian random field (GRF) correction.

## 3 Result

### 3.1 General Clinical Data and Scales

In this study, a total of 41 MwoA patients from middle and high altitude areas and 40 HCs were recruited. However, one participant in HCs group was excluded due to significant expansion of unilateral ventricle volume. Finally, the total number of participants was 41 migraine patients, including 14 males and 27 females, aged 37.34 ± 7.51 (Range 24–50) years old. There were 39 healthy controls, including 15 males and 24 females, aged 35.18 ± 6.62 years (Range 22–48 years).

The scores of SAS, SDS, and PSQI in MwoA group were higher than those in HCs group. However, the differences were not statistically significant (*p* > 0.05). There were no significant differences in years of education, gender and age between two groups (*p* > 0.05). The HIT‐6 and MIDAS scores of the MwoA group were shown in Tables 1 and 2, and the demographic and clinical characteristics of participants in each group were shown in Table 3.

**TABLE 1:** HIT‐6 score of MwoA group in middle and high altitude area.

Fractional interval	Severity	Number of people	Percent(%)
36–49	Have little effect	14	34
50–55	Moderate impact	9	22
56–59	Significant impact	10	24
60–78	Seriously affect	8	20
Total	—	41	100

**Abbreviation**: HIT‐6:Headache Impact Test Scale‐6.

**TABLE 2:** MIDAS score of MwoA group in middle and high altitude area.

Fractional interval	Severity	Number of people	Percent(%)
0–5	Level I	12	29
6–10	Level II	23	56
11–20	Level III	6	15
≥ 21	Level IV	0	0
Total	—	41	100

**Abbreviation**: MIDAS: Migraine Disability Assessment Questionnaire.

**TABLE 3:** General clinical data of MwoA and HCs group at middle and high altitude.

Variables	MwoA	HCs	*p* value
Gender (male/female)	14/27	15/24	0.688^a^
Mean age	37.34 ± 7.51	35.18 ± 6.62	0.238^b^
Years of education	12.0 (7.5, 15.0)	15.0 (9.0, 15.0)	0.568^c^
Disease duration in years	4.15 ± 3.21	—	—
SAS	45.07 ± 10.09	42.95 ± 9.28	0.989^b^
SDS	45.59 ± 9.71	43.49 ± 8.54	0.644^b^
PSQI	11.49 ± 2.95	9.95 ± 3.34	0.400^b^
MIDAS	7.0 (5.0, 10.0)	—	—
HIT‐6	53.37 ± 8.29	—	—

Abbreviations: HCs: Healthy Controls; HIT‐6: Headache Impact Test ‐6; MIDAS: Migraine Disability Assessment questionnaire; MwoA: Patients with migraine without aura; PSQI: Pittsburgh Sleep Quality Index; SAS: Self‐Rating anxiety Scale; SDS: Self‐rating Depression Scale.

^a^ Chi‐square test;

^b^ Two‐sample independent t‐test;

^c^ Rank sum test; when *p* < 0.05, it was considered that there was statistical difference

### 3.2 Group Differences in ALFF

**FIGURE 1:**
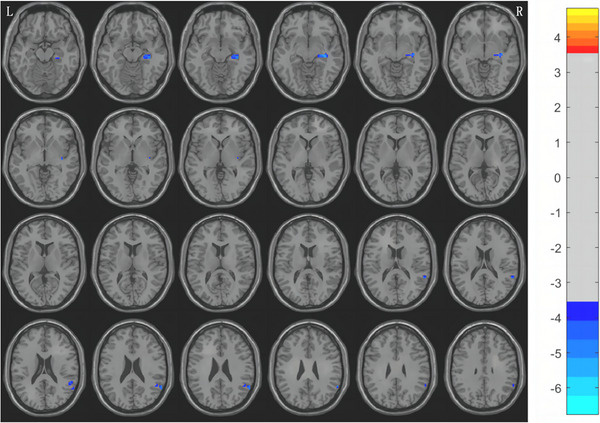
Compared with HCs group, the ALFF value of MwoA group decreased in the right superior temporal gyrus and right hippocampus (GRF corrected, voxel level *p* < 0.001, cluster level *p* < 0.05); L: Left; R: Right; the false bars on the far right represent *t*‐values.

Compared with HCs group, the results showed that the ALFF values of right superior temporal gyrus and right hippocampus in MwoA group at middle and high altitude were decreased (voxel level *p* < 0.001, cluster level *p* < 0.05, GRF corrected) (Figure 1 and Table 4).

**TABLE 4:** The brain regions where there were differences between MwoA and HCs group in ALFF values.

Brain region	Voxel size	MNI coordinate			*T* value	Cohen's *d*
		*x*	*y*	*z*		
Right superior temporal gyrus	20	60	−54	24	−4.914	−0.81
Right hippocampus	28	33	−21	−9	−5.494	−0.90

**Abbreviations**: ALFF: amplitude of low frequency fluctuations; MNI: Montreal Neurological Institute; *T*: Peak intensity.

### 3.3 Group Differences in ReHo

**FIGURE 2:**
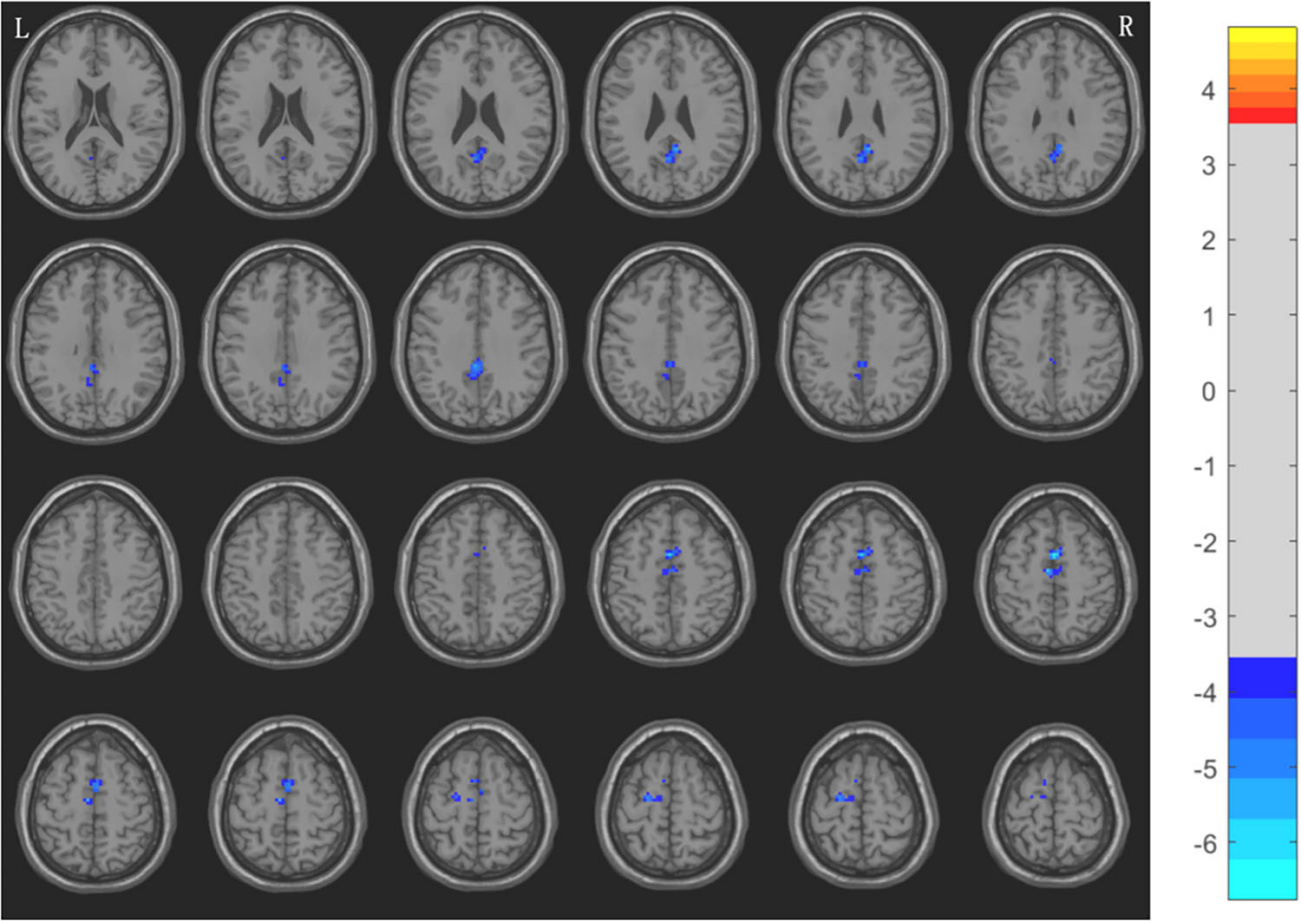
Compared with HCs group, the ReHo value of MwoA group decreased in the bilateral precuneus, bilateral supplementary motor area and the left cingulate gyrus (GRF corrected, voxel level *p* < 0.001, cluster level *p* < 0.05) L: left; R: Right; the false bars on the far right represent *t*‐values.

**FIGURE 3:**
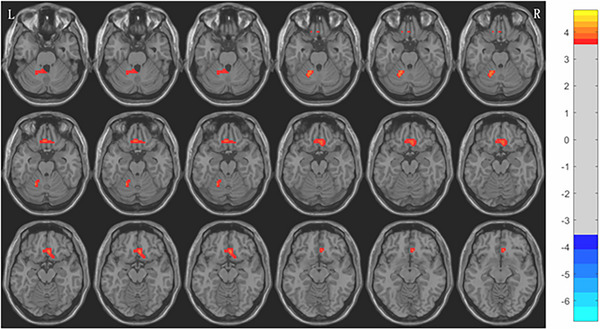
Compared with HCs group, the ReHo value of MwoA group increased in the bilateral rectus gyrus and left cerebellum (voxel level *p* < 0.001, cluster level *p* < 0.05, GRF corrected); L: left; R: Right; The false bars on the far right represent *t*‐values.

Compared with HCs group, the results showed that the ReHo values of bilateral rectus gyrus and left cerebellum in MwoA group at middle and high altitude were significantly increased, while the ReHo values of left cingulate gyrus, bilateral precuneus, and bilateral supplementary motor area were significantly decreased (voxel level *p* < 0.001, cluster level *p* < 0.05, GRF corrected) (Figures 2 and 3, Table 5).

**TABLE 5:** The brain regions where there were differences between MwoA and HCs group in ReHo values.

Brain region	Voxel size	MNI coordinate			*T* value	Cohen's *d*
		*x*	*y*	*z*		
The ReHo value increased (MwoA > HCs)						
Left cerebellum	28	−21	−54	−24	5.438	0.89
Right rectus gyrus	43	6	27	−15	5.144	0.85
Left rectus gyrus	31	−6	28	−19	5.023	0.83
The ReHo value decreases (MwoA < HCs)						
Left cingulate gyrus	32	−3	−36	36	−5.040	−0.83
Right supplementary motor area	22	3	3	53	−5.975	−0.98
Left supplementary motor area	53	0	3	54	−6.355	−1.04
Right precuneus	17	6	−49	26	−6.054	−1.00
Left precuneus	24	−1	−57	27	−5.465	−0.90

*Note*: ALFF: amplitude of low frequency fluctuations; MNI: Montreal Neurological Institute; *T*: Peak intensity.

### 3.4 Correlation Analysis of Imaging Indicators and Clinical Features in MwoA Group

**FIGURE 4:**
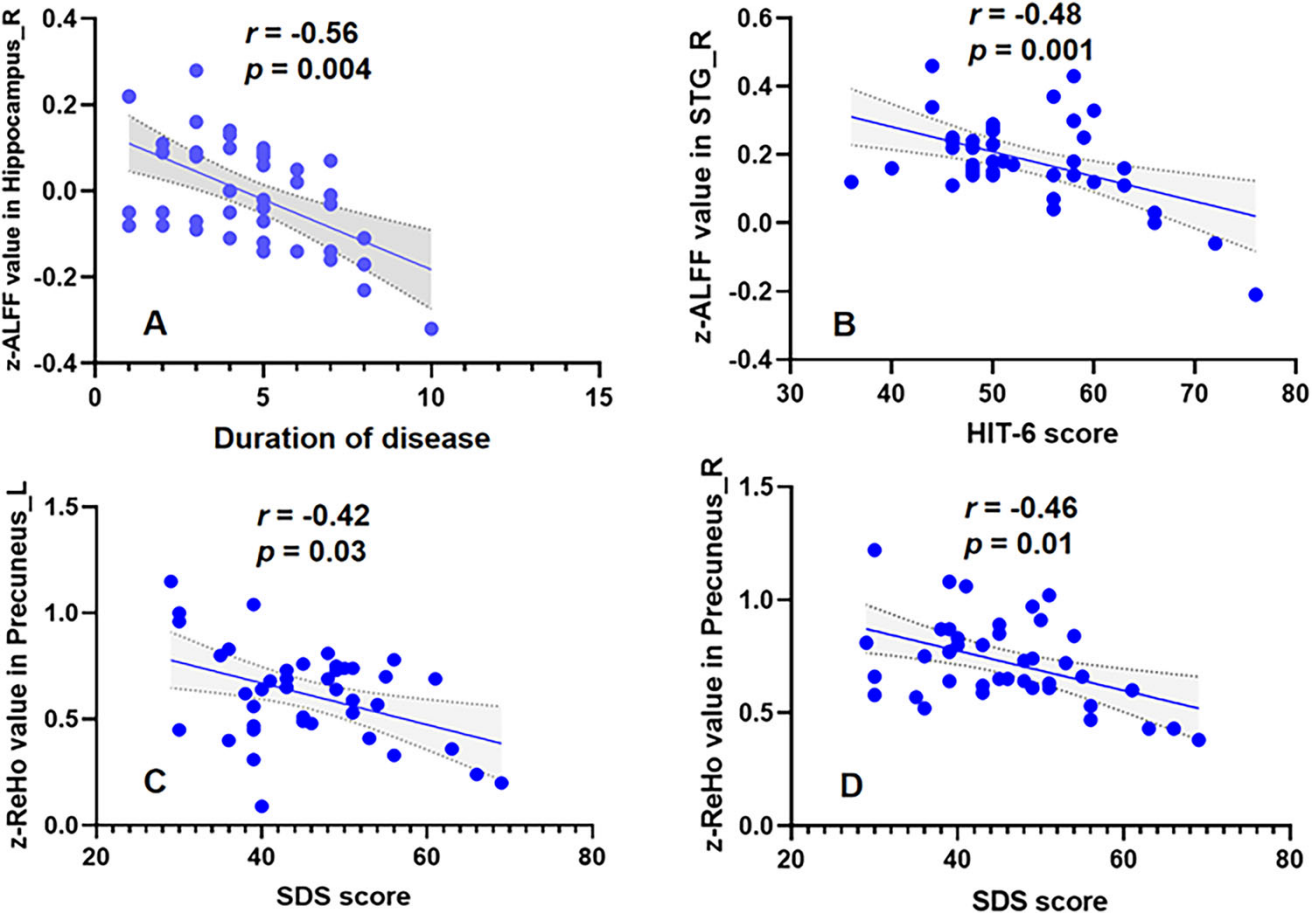
Correlation analysis of imaging indicators and clinical features in MwoA group. The correlation analysis showed that the duration of disease in patients with migraine was negatively correlated with the *z*‐ALFF value of the right hippocampus (*r* = −0.56, *p* = 0.004, Bonferroni correction). The HIT‐6 score was negatively correlated with the *z*‐ALFF value of the right superior temporal gyrus (*r* = −0.48, *p* = 0.001, Bonferroni correction). The SDS score was negatively correlated with the bilateral precuneus *z*‐ReHo values (*r* = −0.42, *p* = 0.03, L; *r* = −0.46, *p* = 0.01, R, Bonferroni correction).

The correlation analysis showed that the duration of disease in patients with migraine was negatively correlated with the *z*‐ALFF value of the right hippocampus(*r* = −0.56, *p* = 0.004, Bonferroni correction, see Figure 4). The HIT‐6 score was negatively correlated with the *z*‐ALFF value of the right superior temporal gyrus (*r* = −0.48, *p* = 0.001, Bonferroni correction, see Figure 4). The SDS score was negatively correlated with the bilateral precuneus *z*‐ReHo values (*r* = −0.42, *p* = 0.03, L; *r* = −0.46, *p* = 0.01, R, Bonferroni correction, see Figure 4).

## 4 Discussion

In this study, we particularly focused on the cerebral functional changes in MwoA patients from mid‐to‐high altitude areas, employing various mental health scales for assessment (Mulder et al. 2003; Linde et al. 2017; Arregui et al. 1991). There were no significant differences in SAS, SDS, and PSQI scales. However, this study found that compared with HCs group, the ALFF values of right superior temporal gyrus and the right hippocampus in MwoA group at mid‐to‐high altitude were decreased; the ReHo values of bilateral rectus gyrus and left cerebellum in MwoA group at mid‐to‐high altitude were significantly increased, while the ReHo values of left cingulate gyrus, bilateral precuneus, and bilateral supplementary motor area were significantly decreased; the correlation analysis showed that the duration of disease in MwoA group was negatively correlated with the *z*‐ALFF value of the right hippocampus; the HIT‐6 score was negatively correlated with the *z*‐ALFF value of the right superior temporal gyrus; the SDS score was negatively correlated with the bilateral precuneus *z*‐ReHo values (Louter et al. 2015; De Angeli et al. 2014).

The rs‐fMRI was used to reveal functional abnormalities in key brain areas and network such as default mode network (DMN) in MwoA patients from mid‐to‐high altitude regions (Messina et al. 2022). The DMN is closely linked to self‐referential thinking, emotional regulation, and pain processing (Ogawa et al. 1990). Our data indicated that MwoA patients from these regions exhibit abnormal activation patterns in the precuneus, posterior cingulate cortex, and superior temporal gyrus, which could be directly associated with the mechanisms of migraine attacks and their disabling symptoms (Lucassen et al. 2014; Schwedt et al. 2014; Kim et al. 2010; Brunklaus et al. 2022).

Particularly, environmental factors in mid‐to‐high altitude regions, such as hypoxia and reduced atmospheric pressure, provided a unique perspective for migraine research (Mulder et al. 2003; Linde et al. 2017). Prolonged exposure to low oxygen could lead to abnormal energy metabolism in brain cells, subsequently affecting brain function and structure, and exacerbating clinical symptoms of migraine (Arregui et al. 1991; Frank et al. 2022). We noted that under hypoxic conditions, brain function changes in MwoA patients might be linked to alterations in cerebral blood supply and metabolism. These physiological changes could influence migraine development and progression through modifications in vascular reactivity and neurotransmitter systems within the brain (Samarasekera 2021).

Although research has explored the mechanisms behind migraine attacks from multiple perspectives, a full understanding remains elusive (Samarasekera 2021). Traditionally thought to be associated with vascular dilation, recent advances in neuroscience have positioned migraine as a primary brain disorder involving neuroinflammation, neurovascular responses, and abnormal cortical excitability. Activation of the trigeminal nerve system and the release of associated neurotransmitters play a crucial role in the pathology, triggering migraine attacks (Lv et al. 2018).

Our imaging findings revealed functional abnormalities in the hippocampus, temporal superior gyrus, and precuneus among patients in mid‐to‐high altitude areas (Schwedt et al. 2014; Liu and Chen 2009; Wen et al. 2023, H. Y. Liu et al. 2018, Wei et al. 2021; Houde et al. 2020; Zhang et al. 2016). Furthermore, the duration of disease in patients with migraine was negatively correlated with the *z*‐ALFF value of the right hippocampus; the HIT‐6 score was negatively correlated with the *z*‐ALFF value of the right superior temporal gyrus; the *z*‐ReHo values of the bilateral precuneus in patients with migraine were significantly correlated with the SDS scores. These regions are crucial for emotional regulation, pain processing, and memory functions. The aberrant brain functions may reflect the difficulties these patients face in managing pain, emotions, and cognitive tasks (Hougaard et al. 2023; Li et al. 2022). Furthermore, the special environmental conditions of mid‐to‐high altitude areas might exacerbate brain function abnormalities by affecting cerebral oxygen saturation and blood flow dynamics.

Moreover, we observed changes in the cerebellum's function among migraine patients in high altitude areas, suggesting a more complex role for the cerebellum in migraine episodes (Ruscheweyh et al. 2014; Wang et al. 2022; Demir et al. 2016; Farago et al. 2017). Known for its involvement in motor control, the cerebellum is also closely related to pain perception, cognition, and emotional processing (Wang et al. 2017, L. Liu et al. 2020; Dai et al. 2022). This finding could provide new targets for migraine treatment strategies in the future.

Although this study has achieved certain results, there are still some limitations. This study's limitations include potential subjectivity in scale assessments, which might affect the objectivity of the results. First, the lack of detailed altitude stratification and inclusion of patients from plain areas, which prevents a direct examination of altitude's impact on MwoA (Dai et al. 2022). Second, although we instructed participants to follow these guidelines, there's no objective standard to measure their actual compliance, thus the quality of functional images may need further evaluation. Third, the study only used a single‐modal fMRI technique to study brain functional activities and lack of other modal fMRI technique (Ogawa et al. 1990). Fourth, this study was a small sample research, and the sample size may have a certain impact on the results. Finally, this study was a cross‐sectional study; it was currently impossible to determine the causal relationship between imaging changes and migraines. Such a relationship would need to be confirmed in future longitudinal studies.

Therefore, future research will expand sample sizes and refine group classifications by different altitudes, employing multimodal fMRI techniques—including structural, functional, and perfusion imaging—to comprehensively examine the central nervous systems of patients. This would uncover the neuroimaging characteristics of MwoA patients in mid‐to‐high altitude environments, providing a more objective imaging basis for future migraine diagnostics and treatment, and advancing migraine research in the world.

## 5 Conclusion

Several brain regions in MwoA patients from mid‐to‐high altitude areas exhibit abnormal spontaneous neural activity through ALFF and ReHo assessments. These brain regions are closely associated with pain processing, cognitive functions, motor control, attention, and emotional regulation. The functional abnormalities in these regions may be relevant to the pathophysiology of MwoA.

## Author Contributions

Jiahui Yao, Qingxin Zhang, Xiaoli Yang, Guocai Yang, Guangyi Yang, and Yuhui Xiong conceived and designed the experiment. Jiahui Yao and Guangyi Yang conducted experiments. Jiahui Yao and Guangyi Yang collected, analyzed, and processed the data. Jiahui Yao, Xiaoli Yang, Qingxin Zhang, and GuangYi Yang wrote the draft, reviewed it, and made revisions. After reading the final text, each author gave their approval.

## Conflicts of Interest

The authors declare no conflicts of interest.

## Funding

This study was supported by the Qinghai Provincial Department of Science and Technology (Grant Number: 2021‐ZJ‐748) and the Health Commission of Qinghai Province (Grant Number: 2024‐wjzdx‐19).

## Peer Review

The peer review history for this article is available at https://publons.com/publon/10.1002/brb3.70975.

## Data Availability

Data will be made available on request.
